# Different material for the SPORT concept brace: short term comparison of Sforzesco and Sibilla brace

**DOI:** 10.1186/1748-7161-8-S1-O39

**Published:** 2013-06-03

**Authors:** F Zaina, S Donzelli, M Lusini, S Negrini

**Affiliations:** 1 ISICO (Italian Scientific Spine Institute), Milan, Italy; 2 Siena University, Siena, Italy; 3 University of Brescia, Brescia, Italy; 4 IRCCS Don Gnocchi, Milan, Italy

## Background

SPoRT braces include the Sforzesco and the Sibilla Brace. The difference in the two consists in the material; Sforzesco is more rigid, and thus used in more severe and rigid curves.

## Aim

To compare the short term results of the Sforzesco and the Sibilla to check the influence of the material.

## Methods

Design: retrospective pre-post study. Protocol: in our database we searched for all patients who were prescribed a Sibilla, or Sforzesco Brace, 21h per day or more for AIS, 30-35° Cobb, more than 10 years old at first evaluation, and no previous brace treatment. We compared data from the last visit before beginning the brace, and the first visit after 6 months of brace treatment. Sforzesco group (SF-G): 78 patients (10 males), age13.6±1.6, 32.9±1.9° Cobb, TRACE 6; 5-8 (median, IQ), ATR 10.8±3° Bunnell, Risser 0.5; 0-2 (median, IQ), BMI 19.24. Sibilla Group (SI-G): 44 patients (5 males), age 12.6±1.6, 32.1±1.6° Cobb, TRACE 6.56; 5.44-7, ATR 9.4±2.9° Bunnell, Risser 2; 1-3, BMI 19.34. Outcome measure: Cobb angle, TRACE, ATR. Statistical Analysis: ANOVA, Chi Square, P<0.05.

## Results

The SF-G had statistically significant larger curves, and larger ATR, more rigid spines, and slightly older, but the difference was not clinically relevant. The average wearing hour for the brace was 20 h for both groups. Both groups improved the Cobb angle (26.4° Sforzesco vs 25.2° Sibilla), TRACE (3.34 vs 4) and ATR (7.6 vs 6.1). No difference was statistically significant. Rigidity, BMI, ATR and initial Risser didn't affect the Cobb correction.

## Conclusion

Both SpoRT braces can improve curves between 30-35° in the short term. No differences have been noticed, so we can conclude that in this population the rigidity of the material doesn't affect the result. It's possible that for larger curves the difference would be more relevant, but we need further studies to verify.
